# Ki-67 Levels and Their Association With Response to Neoadjuvant Chemotherapy in Triple-Negative Breast Cancer: A Prospective Observational Study

**DOI:** 10.7759/cureus.83207

**Published:** 2025-04-29

**Authors:** Ramji Nalla Narendra, Chellappa Vijayakumar, Gothati Haritha, Sahoo Ashok Kumar, Krishnamachari Srinivasan, Dubashi Biswajit, Ashok Badhe Bhawana, Sundar Elangovan

**Affiliations:** 1 Surgery, Rangaraya Medical College, Kakinada, IND; 2 Surgery, Jawaharlal Institute of Postgraduate Medical Education and Research (JIPMER), Puducherry, IND; 3 Radiology, Rangaraya Medical College, Kakinada, IND; 4 Surgery, All India Institute of Medical Sciences (AIIMS), Bhubaneswar, IND; 5 Medical Oncology, Jawaharlal Institute of Postgraduate Medical Education and Research (JIPMER), Puducherry, IND; 6 Pathology, Jawaharlal Institute of Postgraduate Medical Education and Research (JIPMER), Puducherry, IND; 7 Radiology, Jawaharlal Institute of Postgraduate Medical Education and Research (JIPMER), Puducherry, IND

**Keywords:** chemotherapy, ki-67 expression, neoadjuvant chemotherapy, triple-negative

## Abstract

Background

Due to epigenetic changes in the breast cancer gene 1 (*BRCA-1*), triple-negative breast cancers (TNBC) are more responsive to platinum compounds. The prevalence of TNBC across India is found to be 27%-35%. The study was carried out to assess the Ki-67 expression and the response to neoadjuvant chemotherapy (NACT)-docetaxel-carboplatin (DC) regimen in patients with TNBC and to identify the association of Ki-67 expression with the NACT.

Methodology

All newly diagnosed TNBC patients over 18 years of age were subjected to NACT with the DC regimen. Three cycles of NACT had been given at three weekly intervals. The tumor response to NACT was assessed at the end of the third cycle using the response evaluation criteria in solid tumors (RECIST). All patients who completed NACT underwent a modified radical mastectomy (MRM), and the specimen was sent for histopathological examination (HPE). The response to NACT was correlated with the Ki-67 expression. After the surgery, the remaining three cycles of chemotherapy were completed, and adjuvant radiotherapy was given whenever required. The toxicities were documented. The patients were followed until the day of completion of the study.

Results

A total of 23 TNBC patients were included in this study, and the mean Ki-67 level was found to be 36%. There was no significant difference according to age (P = 0.3, 95% CI: -0.15 to 0.45), menopausal status (P = 0.66, 95% CI: -0.24 to 0.38), size (P = 0.22, 95% CI: -0.1 to 0.33), grade (P = 0.33, 95% CI: -0.08 to 0.35), and stage of the tumor (P = 0.17, 95% CI: -0.11 to 0.29) between the partial response and complete response patients. When the Ki-67 level was analyzed in relation to responders and non-responders, tumor size, grade, and nodal status, it was found to be insignificant. When the DC regimen was given to the TNBC patients, it was observed that the complete clinical response, complete imaging response, and complete pathological response were 39.13%, 34.78%, and 47.61%, respectively. The most common side effects of NACT were malaise, nausea, and hair loss. During the follow-up period, 20 patients had no local recurrence or metastatic features.

Conclusion

In the present study, the TNBC had high Ki-67 levels, which were insignificant when compared against age, menopause, tumor size, nodal status, and grade. There was no relation between the partial responder group and the complete response group in terms of age, menopausal status, tumor size, nodal size, and grade. When Ki-67 levels were correlated between responders and non-responders, they were also found to be insignificant. The DC regimen, as NACT, did not have any severe side effects.

## Introduction

Triple-negative breast cancer (TNBC) is known for its aggressive clinical behavior, high recurrence rates, and relatively poor prognosis despite aggressive treatment strategies [1]. The role of Ki-67, a nuclear protein associated with cell proliferation, has garnered attention as a potential marker for predicting treatment response in TNBC. Studies have indicated that high Ki-67 expression is linked to a better pathological complete response (pCR) in TNBC patients undergoing neoadjuvant chemotherapy (NACT), especially with platinum-based regimens [2,3]. Despite these findings, the clinical application of Ki-67 as a predictive marker for treatment outcomes in TNBC remains under exploration, particularly in diverse populations and settings with limited resources [4].

In India, TNBC represents a significant proportion of breast cancer cases, with a prevalence ranging from 27% to 35%. The majority of these cases present as locally advanced breast cancer (LABC). Given the lack of targeted therapies for TNBC, it is crucial to identify reliable biomarkers, such as Ki-67, to guide treatment decisions and improve patient outcomes [5].

This study is novel in its approach to assessing Ki-67 expression in TNBC patients undergoing NACT with the docetaxel-carboplatin regimen (NACT-DC), a commonly used but less studied combination in this context. While prior studies have explored the relationship between Ki-67 and chemotherapy response, this research aims to establish its predictive value specifically for the NACT-DC regimen in a cohort from India, where TNBC is more prevalent. Additionally, by utilizing immunohistochemistry (IHC), which is accessible in settings with limited resources, this study offers a practical approach for resource-constrained environments.

The primary objective of this study is to evaluate the relationship between Ki-67 expression levels and the response to NACT-DC in TNBC patients. We aim to establish whether higher Ki-67 expression correlates with improved chemotherapy response, including pCR. Additionally, this study will assess the feasibility of using Ki-67 as a biomarker for predicting treatment outcomes in TNBC, particularly in resource-limited settings.

## Materials and methods

The study was conducted in a tertiary care hospital in South India from January 2013 to December 2015. The Institute Ethics Committee of Jawaharlal Institute of Postgraduate Medical Education and Research (JIPMER) approved the study (IEC/SC/2012/5/226). It was performed in accordance with the ethical standards set forth by the Declaration of Helsinki. The study was designed as a prospective, observational study.

All consecutive patients with newly diagnosed TNBC by IHC and who were greater than 18 years of age were included. They were preoperatively subjected to non-contrast computed tomography (NCCT) of the thorax for tumor size assessment and subsequently received three cycles of the DC regimen as NACT. Following this, the clinical, radiological, and pathological responses were assessed in these patients. Patients who had been partially treated outside, not fit for DC chemotherapy, and had metastasis were excluded from the study.

Tumor size (T), nodal status (N), metastasis (M), TNM stage, menopausal status, clinical response to NACT, imaging response to NACT, pathological response to NACT, and Ki 67 expression (%) were analyzed. All consecutive patients with clinically diagnosed or suspected breast cancer had undergone trucut biopsy. TNBC patients (defined as negative for estrogen receptor (ER), progesterone receptor (PR), and HER2) were recruited based on the receptor status, which was performed using IHC methods. The Ki-67 expression was estimated using IHC (labeled Cenze Streptavidin Biotin technique) from the same trucut biopsy specimen. All the TNBC patients planned for the NACT-DC regimen were subjected to NCCT to assess the size of the tumor and lymph node. All selected patients were subjected to NACT with docetaxel (75 mg/m²) and carboplatin area under the curve (AUC) [6]. Three cycles of NACT were given at three weekly intervals, and the responses were assessed clinically and radiologically by an NCCT scan. The tumor response to NACT was assessed at the end of the third cycle using the response evaluation criteria in solid tumors (RECIST) [6]. All patients who completed NACT underwent a modified radical mastectomy (MRM), and the specimen was sent for histopathological examination (HPE) for the assessment of the pathological response of the primary tumor and the axillary nodes. The response to NACT was correlated with the Ki-67 expression. After the surgery, the remaining three cycles of chemotherapy were completed, and adjuvant radiotherapy was given whenever required after the completion of chemotherapy. The toxicity was documented according to standard toxicity criteria.

The patients were followed until the day of completion of the study. The ER, PR, and HER2 scoring were performed according to the standard IHC protocols. The scoring system for ER and PR was based on the percentage of positive tumor nuclei, with a cutoff of ≥1% of tumor cells being considered positive for ER or PR expression [6,7]. The toxicity associated with NACT was evaluated using the Common Terminology Criteria for Adverse Events (CTCAE) version 5.0. Toxicities were graded on a scale of 1 to 5, with 1 representing mild symptoms and 5 representing life-threatening adverse events. The most common side effects monitored during the study included nausea, fatigue, hair loss, and hematological toxicity [6,7].

IHC analysis

**Figure 1:**
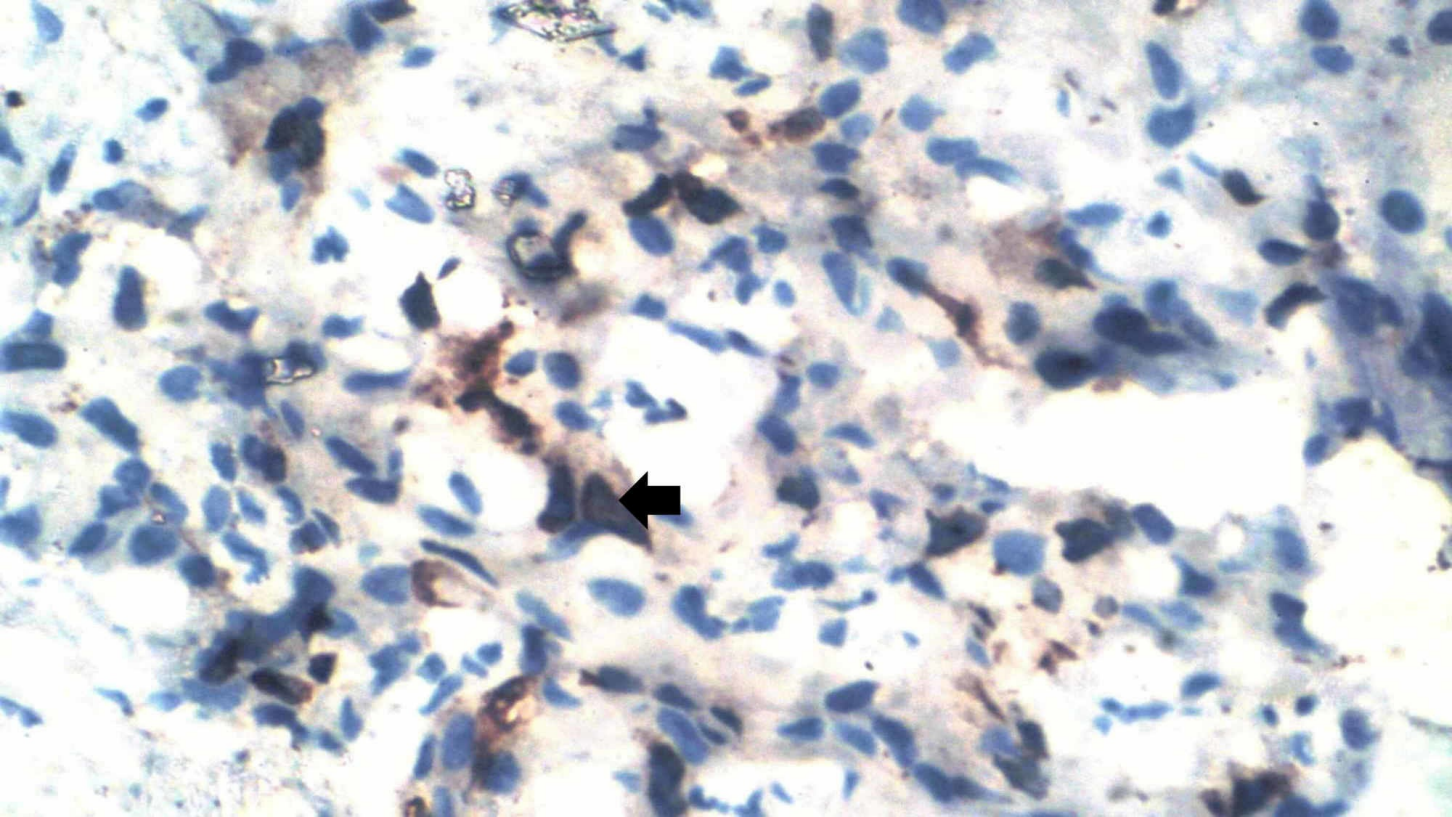
IHC staining of Ki-67: high-power view showing 50% of cells positive for nuclear staining (black arrow) IHC: immunohistochemistry

IHC analysis for the surrogate markers ER, PR, HER2, and Ki-67 was conducted on the formalin-fixed paraffin blocks of the tissue specimen (Figure 1). The tissues that exhibited the antigens were analyzed with the specific antibodies to ascertain the positivity. The immunoreactivity of Ki-67 was measured based on the proportion of positive tumor cells (0%-100%). It was a nuclear antigen and stained using IHC methods with the MIB‑1 antibody on paraffin sections. The St. Gallen International Expert Consensus in 2009 classified levels of Ki-67 as high (≥30%), low (<15%), and intermediate (16%-30%) [8]. In one study, a Ki-67 level greater than 13% was considered high [9]. Similarly, another study determined a cutoff of 13.25% as high, using the receiver operating characteristic method, since Ki-67 is a continuous variable [10]. In another study, the Ki-67 score was defined as the percentage of tumor cells with nuclear staining [11]. They reported that a Ki-67 level of 10%-14% was deemed a high-risk category, associated with poor prognosis. Since most studies take Ki-67 as high when it is nearly 15%, it was decided to take the 15% level as the cutoff in the present study.

Follow-up timeline

Patients were followed at regular intervals after NACT and surgery: (1)⁠ the first follow-up was conducted at four weeks post-chemotherapy for clinical evaluation and assessment of chemotherapy-related adverse events; (2)⁠ subsequent follow-ups were conducted every three months for the first year, which includes clinical examination, imaging (mammography, ultrasound, or NCCT), and tumor marker tests. Patients with relapse or disease progression were referred for further treatment, including adjuvant or palliative care.

Statistical analysis

The collected data were analyzed using GraphPad Instat version 3.06 (Dotmatics, Boston, Massachusetts) and OpenEpi version 2.3.1 software (https://www.openepi.com). To compare continuous variables, the Student's t-test was used for age and tumor size. Fisher's exact test was applied to compare nodal status and menopausal status between the partial response and complete response groups. The relationship between Ki-67 expression and tumor response to chemotherapy was also analyzed using Fisher's exact test. Additionally, the association between disease stage and response to treatment was assessed using the OpenEpi software. All statistical tests were conducted at a 5% significance level (P < 0.05). Given the small sample size (n=23), the power of the statistical analysis may be limited, and results should be interpreted with caution. Future studies with larger sample sizes are needed to validate the findings.

## Results

A total of 563 breast cancer patients were reported during the study period, with 57 (10%) diagnosed with TNBC. Of these, 38 were new cases, while 19 were previously treated elsewhere and confirmed as TNBC by HPE at the current institute. Twenty-three patients met the inclusion criteria for the study. The mean age at presentation was 51.43 years, with 56.13% of patients below 50 years. The distribution of premenopausal and postmenopausal patients was 47.36% and 52.64%, respectively. Among the included patients, 73.68% had LABC (> T3 lesions), while 26.32% had tumors < T3. The mean tumor size was 8.14 cm. The nodal status distribution showed that 57.89% had N1 status, 21.05% had N0, and 10.52% each had N2 and N3. Grade 2 tumors were most common (68.42%), followed by grade 3 (21.05%) and grade 1 (10.52%). The mean Ki-67 level was 36%, with 54.38% of patients having levels >30%, 35.08% between 15% and 30%, and 10.52% having levels <15%.

Most TNBC patients exhibited high Ki-67 levels, but no statistically significant association was found when compared to age, menopausal status, tumor size, nodal status, or grade. Of the 51 patients with high Ki-67 levels, 44 were over 40 years of age, but this was statistically insignificant (p = 1). Similarly, no association was observed between menopausal status and Ki-67 expression, even though 36 postmenopausal patients had high Ki-67 levels (p = 0.66). Tumor size greater than 5 cm was present in 37 patients with high Ki-67, but this also showed no significant correlation (p = 0.3). The association with tumor grade was similarly insignificant (p = 0.3). The mean Ki-67 level in this study was 34.56%, with 91.3% of patients exhibiting levels above 15%. Notably, 52.17% had levels greater than 30%, indicating that high Ki-67 levels are common in TNBC. High Ki-67 levels were observed in 91.3% of T3 lesions, 73.91% of grade 2 tumors, and 65.21% of N1 lesions, suggesting an association with LABC. However, no significant relationship was found between Ki-67 levels and chemotherapy response, with a p-value of 0.94 for partial vs. complete response groups.

A total of four patients were considered for primary surgery: three with T2N0 lesions and one 90-year-old female with a T2N1 lesion, who underwent upfront surgery due to early disease and advanced age. All four patients subsequently received adjuvant anthracycline-based chemotherapy and radiotherapy. Approximately 40.34% of patients were deemed fit for the NACT-DC regimen, with 7% having early-stage carcinomas and 33.33% presenting with LABC. Due to advanced age and reduced cardiac function, 11.5% were deemed unfit for the regimen.

Four patients presented with metastatic disease at diagnosis. One 55-year-old patient with multiple lung, liver, and adrenal metastases was treated with six cycles of the doxorubicin, fluorouracil, and cyclophosphamide regimen (FAC). A 52-year-old patient with liver and bone metastasis received oral capecitabine, zoledronic acid, and palliative radiotherapy. A 65-year-old patient with supraclavicular node metastasis was treated with six cycles of paclitaxel, while another patient with left supraclavicular node metastasis received anthracycline-based chemotherapy.

Of the 14 patients operated on outside, five underwent excision biopsy to confirm the diagnosis, which subsequently revealed early breast carcinoma (EBC). The remaining nine (15.78%) patients had undergone MRM and were referred to the present institute for adjuvant chemotherapy. Five patients had received chemotherapy at a different institution. Three patients were referred after receiving cyclophosphamide, doxorubicin hydrochloride (Adriamycin), and fluorouracil regimen (CAF); one patient was given paclitaxel, and the other patient lost treatment records. All were referred to as non-responding tumors.

When the DC regimen was given to the TNBC patients, it was observed that 39.13% of patients had a complete response clinically, 34.78% of patients showed a complete response imaging-wise, and 47.61% of patients demonstrated a complete pathological response both in the breast and axilla. Additionally, 34.78% of patients exhibited a partial clinical response, 33.33% had a partial response imaging-wise, and 47.61% had a partial tumor response pathologically. Approximately 21.73% of patients had a stable clinical response, and 23.80% had a stable disease imaging-wise (Table 1).

**Table 1:** Different levels of response to docetaxel and carboplatin NACT NACT: neoadjuvant chemotherapy; N: number

Level	Response	N (%)
Clinical (N = 23)	Complete	9 (39.13)
	Partial	8 (34.78)
	Stable	5 (21.73)
	Progression	1 (4.34)
Imaging (N = 21)	Complete	8 (34.78)
	Partial	7 (33.33)
	Stable	5 (23.80)
	Progression	1 (4.76)
Pathological (N = 21)	Complete	10 (47.61)
	Partial	10 (47.61)
	Progression	1 (4.76)

The mean age of patients receiving NACT was 51.65 years, with 78.26% being postmenopausal and 21.73% premenopausal. No significant age difference was observed between the partial response and complete response groups (p = 0.3). Menopausal status also showed no significant difference between the two groups (p = 1).

Regarding tumor size, 96% of patients had tumors larger than 5 cm, while only 4% had tumors between 2 and 5 cm. No significant association was found between tumor size and chemotherapy response (p = 0.22). The majority of patients (78.26%) were at the N1 stage, followed by N0 (7.39%) and N2 (4%), with no significant difference in response by stage (p = 1). Tumor grading revealed that 4% were grade 1, 78.26% grade 2, and 17.39% grade 3. There was no significant difference in response based on tumor grade (p = 0.33) (Table 2).

**Table 2:** Comparison of baseline characters between partial response and complete response groups (n = 21) #: For statistical purposes, N2 is included in N1; *: Student's t-test; **: Fisher's exact test; ***: chi-square test​​​​ SD: standard deviation; CI: confidence interval

Criteria		Partial Responders	Complete Responders	p-value
Mean age		53.81±9.09 SD	50.60±6.93 SD	0.3* 95% CI: -0.15 to 0.45
Menopausal status	Pre-menopausal	2	1	1**
	Post-menopausal	9	9	
	Mean tumor size	9.72±3.06 SD	8.3±1.97 SD	0.22* 95% CI: -0.1 to 0.33
Nodal status^#^	N_0_	2	2	1**
	N_1_	9	8	
Grade	1	1	0	0.33*** 95% CI: -0.08 to 0.35
	2	9	7	
	3	1	3	
Ki-67	<15%	1	1	0.94**
	>15%	10	9	
Stage	Early	2	2	1**
	Late	9	8	

The analysis of early versus late carcinoma response to chemotherapy revealed no significant difference (p = 1). Similarly, the comparison of responses based on tumor stage also showed no significant correlation (p = 0.17) (Table 3).

**Table 3:** Analysis of stage vs. NACT response *: OpenEpi version 2.3.1. Not meeting Cochran's criteria n: number; T: tumor size; N: nodal status; M: metastasis

Response (N=21)	T_3_N_0_M_0_	T_3_N_1_M_0 _& T_3_N_2_M_0_	T_4_N_1_M_0_	p-value
Complete (n=10)	2	7	1	0.17*
Partial (n = 11)	2	4	5	

When the relationship between Ki-67 levels and factors like tumor size, grade, and nodal status was analyzed, it was not significant (Table 4).

**Table 4:** Relationship between Ki-67 index, tumor stage, nodal status, and grade in the NACT-treated group (N = 23) *: Cannot be calculated since, irrespective of Ki 67 status, the tumor size was > 5 cm; **: chi-square test NACT: neoadjuvant chemotherapy; N: number

Parameters		Ki-67 Index			p-value
		<15% (Low)	15-30%	>30%	
Tumor size	<2 cm	0	0	0	Nil*
	2-5 cm	0	0	0	
	>5 cm	2	10	11	
Grade	1	0	0	1	0.7**
	2	2	9	8	
	3	0	1	2	
Nodal	N0	0	2	2	0.67**
	N1	2	6	10	
	N2	0	1	0	

When Ki-67 levels were correlated between responders and non-responders, the p-value was revealed to be statistically insignificant (p = 1) (Table 5).

**Table 5:** Association of Ki-67 in responders and non-responders *: Fisher's exact test N: number

Ki-67 Index	Responders	Non-responders	p-value
<15%	2	0	1*
>15%	18	1	

The most common side effects of NACT were malaise (n = 20), nausea (n = 19), and hair loss (n = 20). Only four patients had febrile neutropenia requiring hospital management. The chemotherapy doses were not reduced in these cases. Only one case showed progressive disease despite chemotherapy. After three cycles of chemotherapy, the tumor completely disappeared in one case, and the patient refused to undergo further treatment. A patient who received chemotherapy demonstrated a partial clinical response. However, this patient got operated on in another facility and was receiving follow-up care at that institution. Both cases were not included in the analysis of the pCR.

Follow-up

A total of 23 patients had received NACT, out of which two patients were lost to follow-up. Of the remaining 21 patients who completed the course of treatment, all were under regular follow-up. The maximum follow-up period was 15 months, and the minimum duration was three months during the study period. A total of 20 patients had no local recurrence or metastatic features. Only one patient, classified as T4b upon presentation, had both local recurrences at the surgical site and distant metastasis to the lungs and bones.

## Discussion

TNBC is known for its aggressive nature and poor prognosis, often presenting in younger individuals with advanced disease [11,12]. This study confirms that TNBC is more common in younger patients with poorly differentiated tumors but reveals differences compared to existing literature. For example, while previous studies have reported TNBC typically in patients under 40, our study found a mean age of 51.43 years, with an even distribution of patients below and above 50 years [12,13]. Additionally, 52.64% of our patients were postmenopausal, contrasting with other studies that found TNBC more common in premenopausal women [14]. Regarding tumor size, our study identified a significantly larger mean size of 8.14 cm compared to smaller sizes found in other studies, suggesting a more advanced disease in our cohort [15,16].

The larger tumor size in our cohort suggests a higher proportion of locally advanced cases, possibly due to delays in diagnosis or a more aggressive disease phenotype. The high rate of T3 and T4 lesions (73.68%) supports this finding, as these stages are linked to a poorer prognosis. The nodal involvement in our study indicates that 57.89% exhibited N1 status and 10.52% each in N2 and N3, suggesting a more advanced disease compared to other studies [17,18]. The higher proportion of grade 3 tumors (21.05%) compared to previous studies may indicate differences in tumor biology or demographics [17,18]. These findings highlight regional or biological factors influencing TNBC presentation and emphasize the need for multicenter studies to validate these results and further explore contributing factors.

Ki-67, a nuclear antigen associated with tumor cell proliferation, is widely used as a marker to assess treatment response in breast cancer [17-19]. Typically, Ki-67 levels in normal breast tissue are below 3%, while in TNBC, these levels are notably higher [20-22]. Our study found a mean Ki-67 expression of 36%, which aligns with previous studies showing elevated Ki-67 levels in TNBC. However, despite this elevated expression, the data did not reveal any significant association between Ki-67 levels and key clinical factors such as age, menopausal status, tumor size, nodal involvement, or tumor grade. This lack of correlation suggests that while Ki-67 is a marker of proliferation, it may not serve as a reliable predictive tool for treatment response in TNBC, at least in the context of our study.

The study also looked at how well the DC regimen works for treating TNBC, since there is no standard chemotherapy plan for this type. The DC regimen has shown promising results in various studies, with response rates varying from 19% to 70% for complete or near pCR. In contrast, our study found a pCR rate of 47.61%, which, while promising, is lower than those found in other reports but consistent with the variability seen in previous trials [23-25]. These differences could reflect factors such as sample size, patient heterogeneity, or variations in chemotherapy administration protocols. Furthermore, while the high complete pathological response rate is encouraging, the lack of clear predictive markers like Ki-67 means that the treatment's effectiveness cannot be confidently predicted based solely on tumor characteristics observed in our cohort. Thus, future studies should focus on integrating additional molecular markers and larger, multicenter trials to further validate the utility of the DC regimen and identify potential biomarkers for predicting treatment response in TNBC.

Although previous studies have linked high Ki-67 levels with improved pathological response rates in TNBC, our study did not find a significant correlation between Ki-67 expression and complete pathological response, possibly due to the small sample size [26,4]. However, tumors with Ki-67 levels above 15% showed a higher overall response rate, though no correlation was found with tumor size, grade, or stage, complicating Ki-67's role as a predictive marker. Notably, 95% of patients responded to NACT, with many showing residual tumors under 4 cm, suggesting potential for breast-conserving surgery (BCS). Our study also found a good correlation between clinical and radiological assessments, emphasizing the need for larger studies to better understand Ki-67's predictive value in TNBC treatment outcomes.

In our study, discrepancies between clinical and radiological assessments were noted, particularly in one patient with stable disease clinically but two distinct masses radiologically, classified as a partial response. Additionally, contrast-enhanced CT (CECT) was more effective than NCCT in differentiating tumor from fibrosis. A patient with progression had a grade 3 lesion with a high-grade ductal carcinoma in situ, potentially suggesting non-*BRCA1*-mutated TNBC, as *BRCA1*-positive TNBC typically responds better to platinum agents. Overall, 95% of patients responded to the DC regimen, with half achieving a complete response and the other half a partial response. Notably, 50% of partial responders had residual tumors small enough for BCS. However, no significant correlation was found between pathological response and tumor size, grade, stage, or Ki-67 levels, likely due to the small sample size. These results suggest that the DC regimen shows promising response rates but requires further investigation in larger studies.

Limitations

The sample size in this study was small, limiting the statistical power to draw definitive conclusions about the NACT response and Ki-67 expression. A larger sample size in future studies is essential for more reliable results. We also did not perform a power analysis, which could have guided sample size determination. The absence of BRCA status evaluation is another limitation, and including it in future studies would provide valuable insights into chemotherapy response. Additionally, as this study was conducted at a single institution, the findings may have limited generalizability, emphasizing the need for multicenter studies.

A more fundamental weakness of this study is the absence of a clear predictive association between Ki-67 and treatment response. Despite our hypothesis, we found no significant correlation between Ki-67 levels and NACT response, which diminishes the impact of the study in establishing Ki-67 as a reliable predictive biomarker. This lack of clear predictive value highlights the need for further investigation with larger sample sizes and more refined methodologies. On the positive side, the prospective design strengthens the reliability of our findings. It enables real-time data collection and minimizes recall bias, which enhances the credibility of the results. This should be emphasized in future publications to underline its significance in supporting the study's relevance.

## Conclusions

In this study, while most TNBC cases exhibited high Ki-67 levels, there was no statistically significant correlation with factors such as age, menopausal status, tumor size, nodal involvement, or grade. Additionally, Ki-67 levels did not differ significantly between partial and complete responders or between responders and non-responders, suggesting Ki-67 alone may not reliably predict chemotherapy response in TNBC.

Although the NACT-DC regimen did not cause severe side effects, a pCR was observed in 47.61% of cases, though not statistically significant. To enhance the study's power, future research should include larger sample sizes and gene expression analysis and explore additional biomarkers to improve understanding and guide personalized therapies for TNBC.
